# The role of Psychological Factors in the Aetiology and Treatment of Vasovagal Syncope

**Published:** 2004-04-01

**Authors:** Jennifer Gracie, Christine Baker, Mark H Freeston, Julia L Newton

**Affiliations:** * Department of Psychology, Royal Victoria Infirmary, Newcastle, NE1 4LP, UK; ¶ Department of Psychology, University of Newcastle, Newcastle, UK; § Falls and Syncope Service, Royal Victoria Infirmary, Newcastle, NE1 4LP, UK

## Introduction

Syncope is a sudden transient loss of consciousness with loss of postural tone, followed by spontaneous recovery [1].  Around 30 percent of the general population have one syncopal event in their lifetime, with 3% having recurrent episodes [2]. Vasovagal syncope (VVS) is an exaggerated tendency towards the common faint that accounts for up to 29% of syncope3 and affects all age groups. VVS is characterised by profound hypotension with or without bradycardia. Those with VVS are at risk of injury during episodes and the long term implications of recurrent episodes of hypotension are unclear [1]. The underlying pathophysiology of VVS is uncertain and current treatments involve salt and fluid replacement and maintenance of blood pressure using mineralocorticoids or alpha agonists [1]. These treatments are largely symptomatic and may be associated with side effects that make their use in younger age groups inappropriate [4].

VVS can generally be differentiated from other causes of syncope through clinical evaluation. A definitive diagnosis of VVS is made by Head-up Tilt Table (HUT) test with the diagnosis of VVS being confirmed when a patient develops hypotension and/or bradycardia in association with syncopal or presyncopal symptoms [5].

One of the main methodological threats to current research into VVS is the appropriate measurement of VVS. A HUT is the most sensitive tool to diagnose VVS [5]. The majority of studies however have recruited participants diagnosed by medical evaluation and symptom pattern recognition only [6-10]. Although this method of diagnosis has some degree of reliability and validity, it is not as reliable as a diagnosis made by HUT.

There can be a wide range of reported illness and disability associated with the same level of disease [11]. The experience of VVS is at best inconvenient, and may be perceived as threatening and disabling.

In this review we will consider the evidence that psychological factors play a role in the development and maintenance of the symptoms of syncope, and more specifically vasovagal syncope, and whether psychological interventions might impact upon the manifestation and consequences of these symptoms.

Research into the psychological factors in syncope has mainly involved groups of participants who have syncope of mixed origin. There are very few studies that have focussed exclusively on VVS as a separate condition. Further, it is also important to appreciate that there are no measures specific to VVS and that in many studies the diagnosis of VVS is by clinical evaluation rather than by a positive diagnostic test, raising the question that all that is syncope may not be vasovagal in origin.

## Psychological factors associated with syncope

### 1. Impact of syncope on reported quality of life

Patients with frequent syncope have been shown to have a markedly reduced quality of life, similar to that of patients with severe rheumatoid arthritis or chronic low back pain [8,12]. This provides some indication of the significantly disabling nature of this condition.

Quality of life appears to decrease with increasing frequency of syncope, and there is evidence of impairment in all reported dimensions - particularly in terms of mobility, usual activities, and self care [13]. This impact upon quality of life is reversible and improves within six months when the frequency of syncope is reduced, as exemplified when syncopal events are reduced on permanent pacemaker implantation [14].

Linzer’s group have developed a disease specific measure of impairment due to recurrent syncope (mixed aetiology) [12]. This tool measures psychosocial impairment due to syncope and has reported difficulties with activities of daily living (71%), driving (60%), physical activities (56%), and even walking (42%). Problems in relationships with friends, family and spouses have also been noted. Twenty one per cent of patients have a high degree of fear and worry about their condition which is not associated with injury due to syncope.

### 2. Psychological reactions to syncope

Patients with syncope have a high prevalence of psychological distress, especially anxiety and depression [6,8,15,16]. Individuals may fear their syncope and the negative consequences of fainting and can be severely disabled by their condition [17].

Insights into thoughts immediately before syncope come from a study involving US Air force active flying personnel who had experienced syncope [9]. Thoughts included predictions about potential bodily or psychological harm, particularly people’s reactions to their action, fear of others laughing at them, or being embarrassed due to being socially inappropriate. Virtually all subjects reported that they felt helpless in avoiding the threatening situation.

Vigilance of somatic symptoms linked with fainting, such as feeling hot and giddy, may also sustain apprehension and fear about fainting. The autonomic symptoms of syncope and anxiety are similar, so fear arousal may amplify the physiological signs associated with syncope and lead to an increase in the fear associated with syncope [18].

 Some studies suggest that co morbid diseases, number of syncopal events, duration of syncope and injury due to syncope have no relationship to impairment due to syncope [12]. Psychological factors may mediate impairment associated with syncope, and this would mirror findings in other fields of chronic disease, for example chronic pain [19] and angina [20]. Studies suggest [6] that psychiatric illness is more likely in females, younger age groups (as would be expected in the general population), and in those with a higher number of previous syncopal events.

Despite the methodological problems that make it difficult to draw definite conclusions for these populations with VVS, there is a suggestion that the ways that people understand their condition and their experience and then make predictions about the consequences of fainting is associated with different styles of coping and adjustment, and subsequent quality of life. This raises some interesting areas of potential psychological investigation.

## Psychological factors associated with vasovagal syncope

Studies specifically carried out in those with VVS are few, and in those with VVS diagnosed exclusively by HUT are rarer still.  It could be argued that it is not appropriate to extrapolate data from heterogeneous groups of syncopal patients to those with VVS.  Our experience would support the fact that VVS can lead to high levels of illness and impact profoundly upon the quality of life of the sufferer.  For example VVS is associated with school absences in children and absence from work in adults [4].

Studies suggest that the measurement of psychological constructs in VVS is narrow and tend to focus on demonstrating there is psychological distress among VVS participants.  The measures used however are not specific to VVS.  For example, McGrady et al [17] used the Beck Depression Inventory (BDI) and the State Trait Anxiety Inventory (STAI).  These give a level of depression and anxiety within the groups, but it does not allow for the question of cause or effect to be resolved.

One retrospective study [16] has gathered data from medical notes around the emotional impact of VVS and the reported stressful aspects of VVS. Over half (56%) reported a history of mood disturbance and twenty-one percent were taking a psychotropic medication. A range of psychological problems were reported including suicidal ideation, but in particular depression, panic and chronic anxiety. This is in line with levels of emotional distress reported in other groups with chronic disease. This study also found that patients often experience disabling and uncomfortable symptoms that persist for hours or days after an episode of syncope. The symptoms they reported as distressing include fatigue, dizziness and dyspnoea.  Over forty percent were anxious, worried and frustrated by their situation. The thoughts reported by this group concerned the condition becoming worse, transportation problems due to driving restrictions, not being able to fulfil occupational or family roles, concern about mood swings, interpersonal relationships and the impact of the condition on spouses.

The more frequently reported personality characteristics of these individuals were of discipline, motivation, organisation, perfectionism, punctuality and sensitivity. In this group, those who struggled most to cope with their condition were those who tended to be somewhat self-stressing in their personal style.

The perceived negative consequences of fainting and assumptions of having little or no control together may result in fear and avoidance or restriction of activity leads to an impact on the individual’s quality of life. In anxiety disorder such as panic, avoidance and other self-protecting behaviours have been shown to reinforce the belief that particular situations are dangerous and prevent the development of more adaptive ways of coping with the condition [21].

## The potential role of psychological intervention in the treatment of VVS

Given the high prevalence of psychological dysfunction in those with syncope and VVS and the role of illness-related beliefs and predictions in this condition, psychological interventions could potentially have a part to play in the management of VVS. Indeed, case reports have described the successful use of applied tension (the use of physical manoeuvres such as muscle tensing) and cognitive behaviour therapy (CBT) in patients with VVS [22-26].

A recent case series of patients with a definite diagnosis of VVS (i.e. positive head up tilt and full reproduction of symptoms), in whom conventional treatments had not improved symptoms showed that CBT (incorporating applied tension) resulted in dramatic improvements in symptomatology, consultation behaviour and outcome.  In nine patients there were significant reductions in syncopal episodes and consultation rates post CBT intervention with clear subjective improvements in quality of life and ability to return to work or school [4].  The CBT treatment components included identifying and restructuring unhelpful beliefs; addressing maladaptive somatic attention; reducing avoidance of certain activities and situations; the use of applied tension, and addressing idiosyncratic issues, for example, difficulties sleeping and coping with reactions of others.

Whether similar affects are seen in patients with milder symptoms of VVS is unclear. Likewise, whether psychological symptoms are present early in the natural history of the disease or whether psychological symptoms develop as the symptoms become more entrenched requires clarifying.

There is evolving evidence that the SSRI class of antidepressant is effective in the treatment of VVS and the neurotransmitter serotonin has been implicated in the aetiology of VVS. Serotonin inhibits central neurons that regulate sympathetic activity and there is an association between VVS and depression, in which serotonin is also involved [27]. Further work is needed in order to clarify whether the effectiveness of SSRI in VVS is due to syncope recurrence or whether it has affects upon the subjects’ mood and ability to cope with their symptoms.

## Conclusions

There are a number of psychological issues that appear to be important in the development and maintenance of disability and psychological distress associated with VVS. Current research suggests there is a role for psychological intervention in addressing the distress and disability caused by VVS. Further longitudinal research may help to develop the understanding of the experience of adapting to and coping with VVS, and provide a valuable insight into the long-term psychological consequences of having VVS. Researchers must aim to study pure groups of patients with VVS and develop and use measures that are specific to VVS. This process has started with the development of a measure of impairment due to syncope [8,12]. More work is needed to identify and match effective interventions to those who might most benefit.
